# Affordability and features of home scales for self-weighing

**DOI:** 10.1111/cob.12475

**Published:** 2021-06-30

**Authors:** Ji Seok Park, Carolyn Bramante, Rachit Vakil, Grace Lee, Kimberly Gudzune

**Affiliations:** 1 Digestive Disease and Surgery Institute, Cleveland Clinic, Cleveland, Ohio, USA; 2 Johns Hopkins Bloomberg School of Public Health, Baltimore, Maryland, USA; 3 Division of General Internal Medicine, University of Minnesota Medical School, Minneapolis, Minnesota, USA; 4 Division of General Internal Medicine, Johns Hopkins Hospital, Baltimore, Maryland, USA; 5 Heart and Vascular Institute, Johns Hopkins Hospital, Baltimore, Maryland, USA

**Keywords:** medical equipment, obesity, self-weighing

## Abstract

Self-weighing is an evidence-based weight management strategy, which requires patients to have a home scale. For clinicians to effectively counsel patients on self-weighing, they should be aware of the costs and features available in typical home scales. Our objective was to describe the cost and features of the top bathroom scales available online. We performed content analysis of top 100 scales listed on a popular online retailer. Two coders independently extracted price and scale features (i.e., digital connectivity, body mass index [BMI] calculation, maximum weight accommodated). We used *t*-tests and ANOVA, as appropriate, to examine the relationships between price and features. Among the 97 scales included, mean scale price was $28.99 (SD $21.06; range $7.20–$139.95). Of the advanced features, 20.6% of scales had digital connectivity and 28.9% calculated BMI. Scales with advanced features cost significantly more than scales without (digital connectivity: $49.18 vs. $23.74, *P* < 0.001; BMI calculation: $42.92 vs. $23.33, *P* < 0.001). Most scales (76.2%) had a maximum weight of 351–400 lbs, and only 17.5% could accommodate >400 lbs. Price was higher for scales with a higher maximum weight (*P* = 0.002). No scale with maximum weight > 500 lbs had advanced features. Scales that have digital connectivity for telemedicine or can accommodate higher weights are less commonly available online and their costs may be prohibitive for some patients who need these features. Future research might consider testing whether insurance coverage for scales improves scale access and patient weight management outcomes.

## 1 | INTRODUCTION

Individuals with overweight or obesity have increased risk of cardiovascular disease, type 2 diabetes and several forms of cancer.^1–6^ Sustained weight loss of as little as 3–5% can produce clinically meaningful reductions in cardiometabolic risk factors and reduces the risk of developing type 2 diabetes.^7^ In most of these behavioural weight-loss interventions, the intervention includes three major components—dietary modification, increased physical activity, and behavioural strategies to promote and sustain lifestyle change.^7^

Self-monitoring is an evidence-based behavioural weight-loss strategy grounded in behaviour change theory, which includes tracking weight.^8^ Self-weighing promotes weight loss by helping individuals catch gains in weight and alert them to alter behaviours before the weight gain becomes significant.^9^ Self-weighing is more effective for weight loss than self-monitoring energy intake or activity among adults,^9–16^ and adherence with self-weighing is generally higher than for monitoring these other components.^17–19^ More frequent self-weighing, such as daily weighing, results in better weight loss and weight loss maintenance.^11,12^

Having a scale in the home may increase feasibility of daily weighing. Industry standards for accuracy exist for home scale manufacturers^20,21^ and prior research has shown that digital home scales are generally accurate.^22^ To identify accurate scales, patients may rely upon assessments from consumer associations—some reports are freely available, while others may charge a fee to access.^23,24^ A recent study showed that most primary care patients are willing to self-weigh and to own a scale.^25^ Home scales may facilitate the primary care clinician’s ability to support patients between clinic visits—whether self-weighing is indicated for weight loss to treat obesity or for routine monitoring as part of heart failure management. However, buying a scale can be financially challenging for patients, particularly for low-income individuals.^25,26^ No broad insurance coverage currently exists to cover home scales as durable medical equipment; therefore, patients incur all costs. To date, no studies have described the typical costs to buy a home scale—an essential component for clinicians to discuss when counselling patients.

The COVID-19 pandemic has demonstrated another role of home scales for primary care delivered via telemedicine—patients self-weighing to provide a vital sign. In this setting, digital connectivity technologies (e.g., WiFi, Bluetooth) to transmit weights to the clinic can be invaluable for clinical care. However, patients would have to purchase a scale with such technology. Another important scale feature for clinical care is the weight maximum, as patients need to ensure that the scale can accommodate their weight. Other accessibility features such as a wall-mount display or audio function may help patients use scales who have disabilities (e.g., visual impairment). To date, no studies have determined the prevalence of scales with these features and whether such features are associated with increased scale costs.

For clinicians to effectively counsel patients on self-weighing as part of clinical care, they need to be aware of the costs and features available in typical home scales to help problem-solve with their patients. Therefore, our objectives were to describe the cost and features of best-selling home scales available online, and to understand the relationship between features and costs. We hypothesized that presence of digital connectivity and higher maximum weight would be associated with higher costs as compared to scales without these features.

## 2 | METHODS

In this descriptive study, we evaluated the price and features of the top 100 best-selling body weight scales on the online retailer, Amazon.com. The Johns Hopkins School of Medicine Institutional Review Board approved this study.

### 2.1 | Data source

We identified the top 100 best-selling body weight scales using the “top sellers” list generated by the online retailer. Scales were excluded if they were marketed as baby or pet scales (*n* = 3). We captured images of each webpage from the online retailer that featured theses scales on 9 July 2018 between 6 and 7 pm Eastern Time.

We performed a content analysis of all webpages to generate quantitative data for analysis. Prior studies have used content analysis to examine portrayals of obesity in the U.S. media abstract information from community-based weight-loss programmes,^27–30^ and we adapted these strategies for this study. We developed a coding scheme to extract relevant content including cost as well as the following scale features: digital connectivity (defined as WiFi, cellular or Bluetooth technologies), body fat analysis, body mass index (BMI) calculation, maximum weight, weight increment, power source, location of display, and audio function. We considered “advanced features” to be digital connectivity, body fat analysis, and BMI calculation. Two team members independently reviewed each page and extracted information from text and images. Discrepancies between the two coders were resolved through consensus between reviewers or adjudication by a senior reviewer if consensus was not reached.

### 2.2 | Variables

We examined scale costs as a continuous variable, and we also scaled cost in $5 increments to create meaningful magnitudes of change to determine distribution of costs. We examined each advanced feature as separate dichotomous variables. We also created a dichotomous variable to identify scales with two or more advanced features. We examined maximum weight as a categorical variable by grouping weight in 50 lbs increments (range of groups: 301–350 lbs to 501–550 lbs). All other scale features were dichotomous variables.

### 2.3 | Analyses

Data were analysed with Stata (Stata/IC version 16: STATA Corp LLC., College Station, TX). We conducted descriptive analyses of all variables. We used *t*-tests and ANOVA, as appropriate, to examine the relationship of price with presence of advanced features and weight maximum.

## 3 | RESULTS

We included a total of 97 scales in the study. Table 1 describes the characteristics of included scales.

**FIGURE 1:**
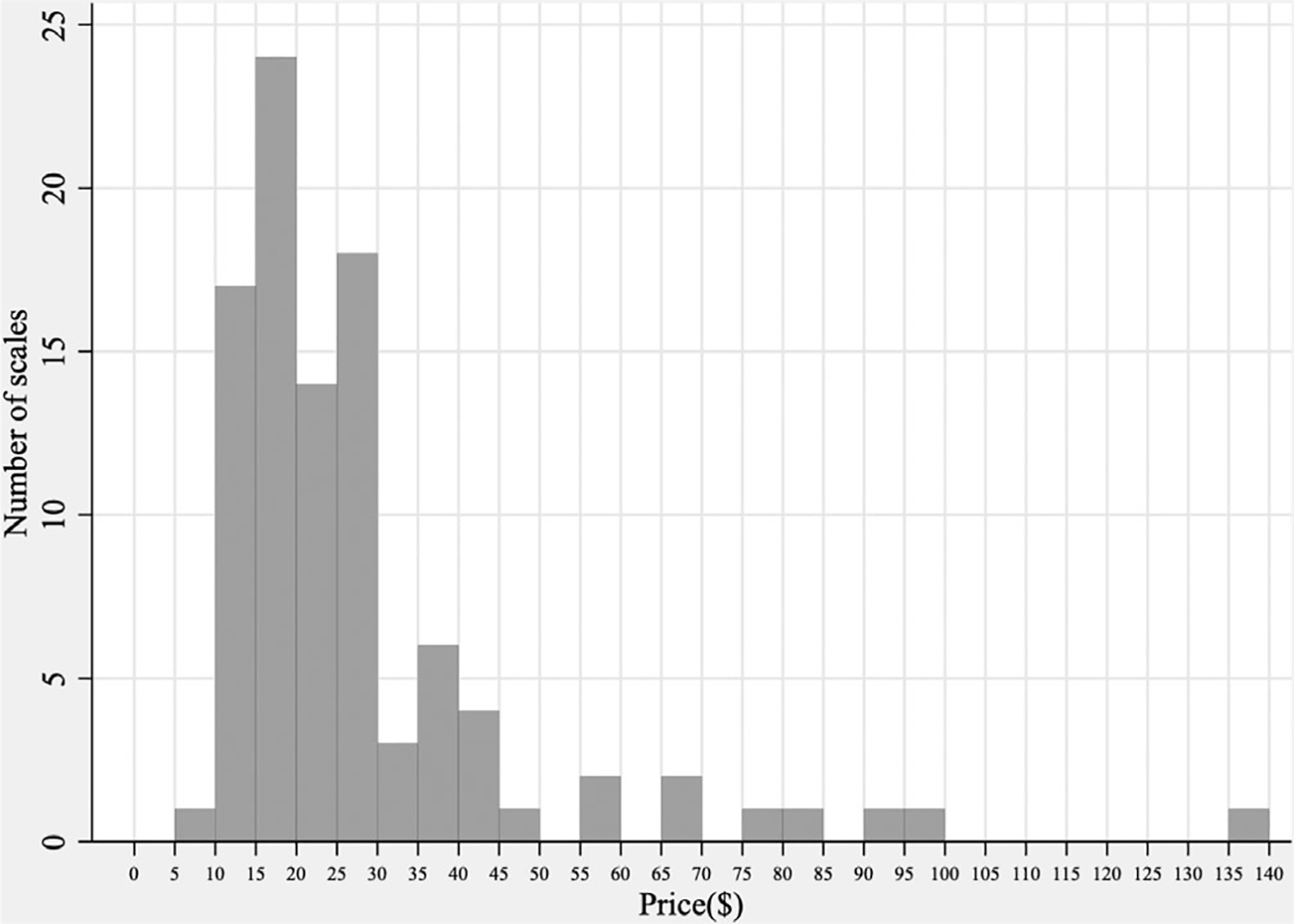
Distribution of costs of best-selling scales on a popular online retailer in 2018 (*n* = 97)

Overall, mean cost was $28.99 (SD $21.06), and costs ranged from range $7.20 to $139.95. Figure 1 shows the distribution of scale costs—24.7% cost between $15 and $20, which was the most common price range. The distribution of costs was right skewed with expensive outliers (Figure 1).

**FIGURE 2:**
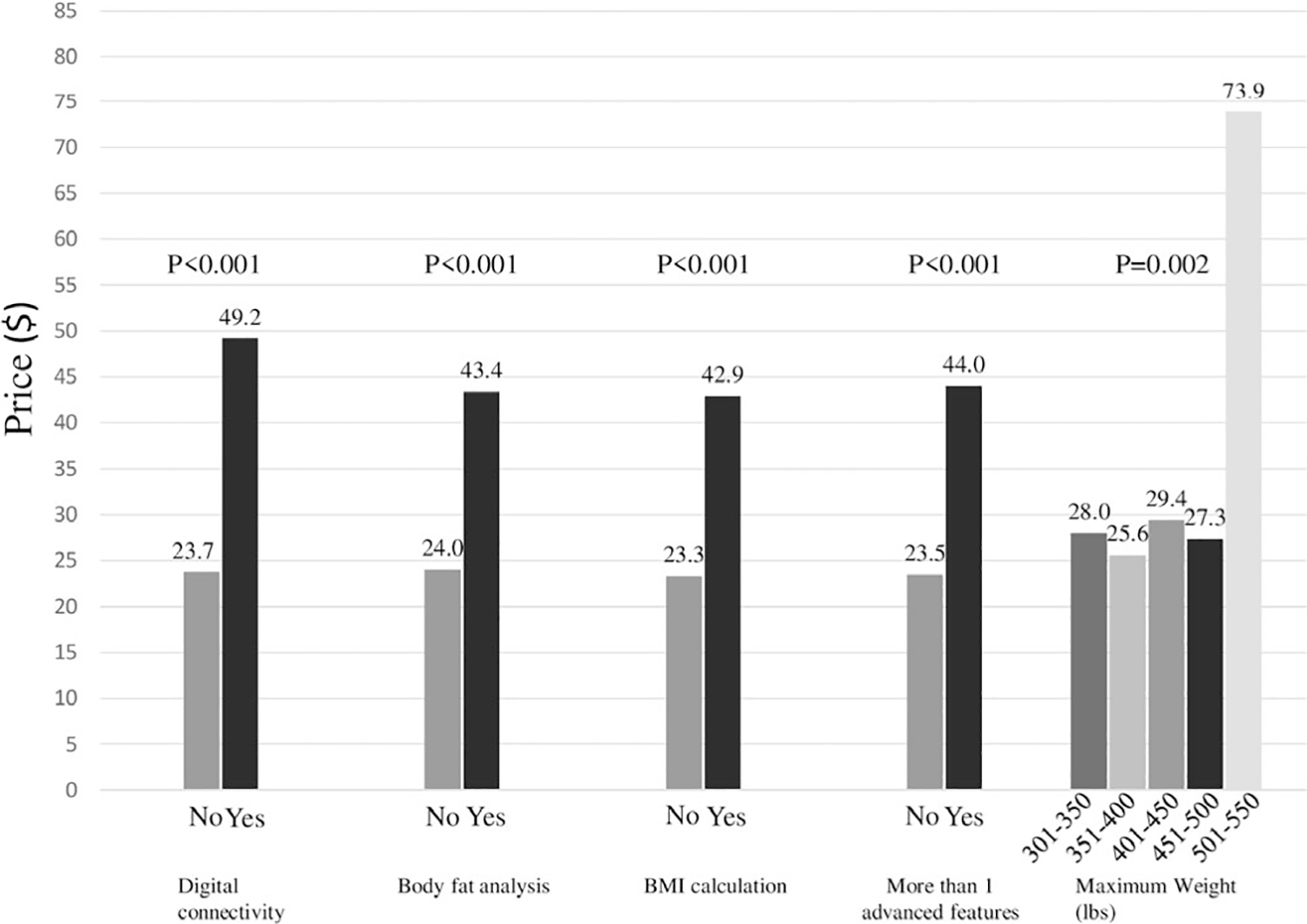
Comparison 2018 costs of best-selling scales on a popular online retailer by presence of advanced features and by weight maximum (*n* = 97), *P*-values calculated with *t*-tests or ANOVA, as appropriate

Regarding advanced features, 20.6% had digital connectivity, 25.8% had body fat analysis, and 28.9% calculated BMI (Table 1). We identified that 26.8% had more than one advanced feature. Scales with advanced features cost significantly more than scales without these features (Figure 2) (digital connectivity: $49.18 vs. $23.74, *P* < 0.001; body fat analysis: $43.35 vs. $24.00, *P* < 0.001; BMI calculation: $42.92 vs. $23.33, *P* < 0.001). Mean price for scales with more than one advanced feature was $43.99 as compared to $23.49 for scales with 0–1 (*P* < 0.001).

When examining maximum weight capacity, most scales (80.4%) had a maximum weight of 351–400 lbs (Table 1), and only 18.5% could measure more than 400 lbs. The highest maximum weight identified was 550 lbs. Scales accommodating higher weights also had significantly higher costs (*P* = 0.002) (Figure 2). Very few scales had other features such as wall mount display or audio function (Table 1).

## 4 | DISCUSSION

To our knowledge, this is the first study to report costs and features of home scales available from a popular online retailer. We found that the average cost of best-selling home scales was approximately $29. Most scales did not offer digital connectivity technologies. In addition, few scales could accommodate patients over 400 lbs and very few offered features like audio function that can be key for patients with certain disabilities. Patients who need these features have limited options from which to choose. In contrast, features like body fat analysis and BMI calculation were available in over 25% of scales. The utility of these measures as part of self-weighing is unclear, and assessments of body composition through bioelectrical impedance analysis (BIA) may be subject to measurement error.^31^

We also found that scales with features such as digital connectivity, body fat analysis, BMI calculation, and ability to accommodate greater body weights were more expensive. Given that prior research has identified cost as a common barrier for low-income patients in accessing scales,^25,26^ these patients who would like to participate in telemedicine using digital connectivity or who have higher body weights may have difficulties finding a scale that they can afford with the features that they need. In contrast, clinicians could advise patients that they do not need to pay more for a scale that has body fat analysis and BMI calculation, as these estimates do not have a clear role in self-weighing.

Since self-weighing is an evidence-based and effective weight management strategy,^9,12,14^ identifying and testing strategies that could address the financial barriers to obtaining a scale are needed. One promising solution could be insurance coverage for scales, as buying a scale can be economically challenging for patients. There is precedence for insurance coverage of durable medical equipment, such as blood pressure cuffs, glucometers, continuous positive airway pressure devices, nebulizers, wheel-chairs, and oxygen equipment.^32^ Clinicians could apply this existing infrastructure for prescribing durable medical equipment to scales, so that these devices could be delivered to patients’ homes or available at pharmacies. Previously, the Centers for Medicare & Medicaid Services (CMS) extended Medicare coverage of ambulatory blood pressure monitoring devices in response to a formal request for widened coverage determination from medical organizations including the American Heart Association and American Medical Association.^33^ The results of this study may contribute to the evidence to support future efforts by organizations such as The Obesity Society or the American Board of Obesity Medicine to lobby CMS to extend this coverage to Medicare beneficiaries. Given the current COVID-19 pandemic, organizations outside the obesity field might also join such an effort in order to improve remote telemedicine care delivery by have a home scale available to capture patient weight.

We also would like to highlight some other potential challenges that patients may face when purchasing a scale. First, we noted that stating maximum weight capacity was not required by the online retailer, as 5.2% of scales’ webpages did not contain this information. Failure to provide this information could make identifying an appropriate scale challenging for patients, particularly patients with class 3 obesity. Second, very few scales offered features adapted to disabled populations—audio function so patients with visual impairment can hear their weight or a wall mounted display to make accurately reading the output for patients with physical challenges or mobility issues. Lack of these adaptive designs may impair these populations engagement in weight management and comprehensive telemedicine services.

Our study has several limitations. While we captured data from a popular online retailer, we did not evaluate costs outcomes for different online retailers and brick-and-mortar stores. While prices may be different, we suspect that the available number of products through these other vendors is likely to be limited. We captured webpages on a single day in 2018, and prices and presences of features and maximum weight capacities may have changed since this time. We relied upon the online retailer’s algorithm to identify their 100 best-selling scales—we did not have a way to confirm this algorithm. Limiting to the 100 best-selling scales does not capture all possible scales available from the retailer. We did not assess accuracy of the scales—clinicians should be aware that industry standards for accuracy exist for home scale manufacturers^20,21^ and prior research has shown that digital home scales are generally accurate.^22^ Finally, we did not have a way to capture sales data or information about consumers purchasing the items.

In conclusion, this study provides important understanding of the price and available features of the home scales available from a popular online retailer. Scales with advanced features, such as digital connectivity, body fat analysis, and BMI calculation, were more expensive. Scales that can accommodate higher weight were also more expensive, which may limit options for patients with class 3 obesity. In the future, insurance coverage for home scales could be a strategy explored to reduce cost as a barrier to scale access.

## Figures and Tables

**TABLE 1:** Characteristics of top-selling scales on a popular online retailer in 2018

Characteristics	Sample (*n* = 97)
Costs	
Mean cost in US$ (SD)	$28.99 ($21.06)
Advanced features	
Digital connectivity	20.6%
Body fat analysis	25.8%
BMI calculation	28.9%
More than one advanced feature	26.8%
Weight features	
Weight maximum groups	
301–350 lbs	1.0%
351–400 lbs	76.2%
401–450 lbs	13.4%
451–500 lbs	1.0%
501–550 lbs	3.1%
Missing^a^	5.2%
Weight increment of 0.2 lbs	66%
Other features	
Power source	
Alkaline batteries	86.6%
Lithium battery	13.4%
Wall mount display	1.0%
Audio function	2.1%

Abbreviations: BMI, body mass index.

a Maximum weight not reported in the product description.

## What is already known about this subject?

- Cost is a barrier for patients owning a scale for self-monitoring of weight.
- Self-weighing improves weight management.

## What this study adds?

- Scales with digital connectivity or ability to accommodate greater body weights are less common and cost significantly more than scales without these features.
- We identified no scales that offered both of these features among the top-selling options online.
- Clinicians should be aware that scale features and scale costs may be a barrier to self-weighing for patients.
